# Ten-year clinical performance of non-precious metal double crowns with friction pins in severely reduced dentitions—a retrospective study

**DOI:** 10.1007/s00784-022-04788-0

**Published:** 2022-11-21

**Authors:** Sebastian Hinz, Wolfgang Bömicke, Ramona Schweyen, Tobias Bensel

**Affiliations:** 1 Department of Prosthetic Dentistry, University School of Dental Medicine, Martin-Luther-University Halle-Wittenberg, Magdeburger Str. 16, 06112 Halle, Germany; 2 Department of Prosthetic Dentistry, University Hospital Heidelberg, University of Heidelberg, Im Neuenheimer Feld 400, 69120 Heidelberg, Germany; 3 Private Practice, Zahnarztpraxis Am Rain, Am Rain 2, 04178 Leipzig, Germany

**Keywords:** Partial denture, Double crowns, Friction pins, Survival rate, Severely reduced dentition

## Abstract

**Objectives:**

This follow-up study aimed at collecting long-term data for removable partial dentures (RPDs) retained by double crowns with spark-eroded friction pins (DCP) and comparing them in the presence of severely reduced dentition (SRD) and non-SRD (NSRD, i.e. residual dentition with more than three abutment teeth) after a 10-year wearing period.

**Materials and methods:**

A total of 158 participants (*n* = 71, 44.9% women) aged 62.5 ± 12.7 years with 182 prostheses on 520 abutment teeth were followed up between 2006 and 2022. The SRD group included 144 RPDs supported by 314 abutment teeth. The data collection was performed retrospectively. 10-year survival rates of RPDs and abutment teeth were determined using the Kaplan–Meier method and compared using the log-rank test for SRD and NSRD, among others. Cox regression analyses were conducted to isolate risk factors for the survival of both RPDs and abutment teeth.

**Results:**

The 10-year cumulative survival rate of all abutment teeth was 65.6% with significantly lower values in the SRD group (53.5%) (*p* < 0.001). The survival rate for all RPDs was 65.5%. The SRD group showed lower survival rates (57.9%) (*p* = 0.004). The number and location of the abutment teeth had a significant influence on the survival rates of the RPDs and the abutment teeth. Age, sex, jaw, relining, and vitality had a significant influence on the abutment teeth survival rates.

**Conclusions:**

RPDs showed an acceptable clinical survival rate after 10 years. The number, location, and vitality of abutment teeth were factors that influenced the survival of both RPDs and abutment teeth.

**Clinical relevance:**

Consideration of the influencing factors found can help improve the prognostic assessment of double crown-retained dentures in the context of prosthetic therapy planning.

## Introduction

Double crowns serve as attachments to removable partial dentures (RPDs). They are a universal connecting element and can be used with abutment teeth as well as implants [1, 2]. Double crowns transfer forces acting on the denture rigidly and directly to the abutments and, for tooth-supported prostheses, represent an alternative to cast clasps [3–6].

In general, double crowns consist of a primary and a secondary crown [7]. The primary crown has the shape of a coping and is fixed to the abutment tooth or the implant abutment. For implant-supported prostheses, the abutment itself may also have the shape of the coping. The secondary crown fits precisely over the primary crown and is part of the RPD framework. Sub-types exist that differ in terms of the materials used for fabrication and on how retention is created between the primary and secondary crowns [3, 4, 7–18]. Regardless of the residual dentition (number of potential abutment teeth), double crowns can be used universally. They have been proven successful in cases with reduced residual dentition (less than 4 teeth) and unfavorable distribution of abutment teeth, as well as in situations with high residual dentition (more than 4 teeth) and favorable distribution of abutment teeth [6, 19–22].

In the severely reduced dentition (SRD), less than 4 teeth are available for supporting an RPD [23]. This can result in the prosthesis being subjected to unfavorable leverage forces during function, causing tilting of the denture and unphysiological loading of the abutments [23–25]. This problem may be further exacerbated as the number of abutments decreases [26]. Additionally, the number and distribution of the residual teeth consequently influence the long-term success of the entire denture [27].

The most commonly used types of double crowns are conical crowns with conical side walls and telescopic crowns with parallel side walls [19–21, 28]. Double crowns with additional retention elements are also well known, but less frequently used. Both telescopic and conical crowns create retention by static friction between the primary and secondary crown [4]. Double crowns with additional retention elements, on the other hand, feature a clearance fit between the primary and secondary crown. Retention is generated exclusively by the retention element. The Marburg double crown is the most common double crown system that includes an additional retention element. Primary and secondary crowns are made of a cobalt-chromium-molybdenum (Co-Cr–Mo) alloy with an additional retention element within the secondary crown, which is spherically elastically fixed with a corresponding recess in the primary crown (TC.SNAP, Si-tec, Herdecke, Germany) [5, 29, 30].

Double crowns with spark-eroded friction pins (DCP) investigated in this study represent another variant [23]. Here, the primary and secondary crowns are manufactured with a tension-free seat and no inherent retention. The retention is achieved with the friction pin, which tensions when the secondary crown slides onto the primary crown. In this type of double crown, the secondary crowns are part of the denture’s framework, therefore a tertiary structure is not necessary. All parts are made from the same Co-Cr–Mo alloy. This is characterized by its high biocompatibility and low allergenic potential [31–35]. An elastically supported retention element, such as in the Marburg double crown, is not necessary with this design.

Overall, denture retention has a great impact on patient satisfaction, which contributes to the acceptance of the dentures and thus contributes to the success of the therapy [36].

Various studies have dealt with retention-force losses of different types of double crowns [3, 4, 37–46]. The retention force decreases during the wearing period of nearly all types of double crowns. In the case of telescopic and conical crowns, the loss of retention force can only be compensated by renewing the entire denture. Double crowns with additional retention elements are advantageous since the retention is created using prefabricated components. In the double crowns with friction pins investigated in this study, it is possible to exchange, reactivate, and adjust retention forces according to patient preference [4, 19, 23].

Previously, double-crown prostheses with spark-eroded friction pins have been proven clinically successful within a 5-year follow-up period [23]. Additionally, the survival rates were similar to those of other double crown systems. The present study aimed to determine the 10-year survival of both RPDs retained by a double crown with spark-eroded friction pins and the abutment teeth and to evaluate whether there is a difference between RPDs in SRD and non-SRD (NSRD, residual dentition with more than 3 abutment teeth) cases. Furthermore, a comprehensive study should also examine the effects of the participant’s age, sex, jaw, number, and location of abutment teeth, as well as abutment tooth vitality on both RPDs and abutment teeth’s survival.

As null hypotheses, it was expected that the 10-year survival rates of both RPDs and abutment teeth would be equal in SRDs and NSRDs and that the above-mentioned variables have no effect on their survival.

## Materials and methods

### Participants

The study considered 158 participants (*n* = 71, 44.9% women) in whom 182 RPDs were placed on 520 abutment teeth. On the day of placement, the mean age of the participants was 62.6 ± 12.7 years (range, 24.5 to 87.0). The mean observation period was 67.2 ± 39.7 months (range, 1.4 to 158.8). The observation period was from January 2006 to January 2022 and all RPDs were provided and followed up at the Department for Prosthodontics at the Martin-Luther-University Halle-Wittenberg.

The study protocol of this present follow-up study was approved by the Ethics Committee of the Medical Faculty of the Martin Luther University Halle-Wittenberg (Registration No.: 2016–129) and complies with the Declaration of Helsinki on the Ethical Principles of Medical Research.

### Pretreatment

All participants were thoroughly clinically examined and screened in accordance with the clinical guidelines of the Department of Prosthodontics at Martin Luther University. If conservative or periodontal pretreatment was required, it was performed accordingly.

### Inclusion criteria

Only adult participants treated with non-precious metal double crowns with friction pins on all remaining teeth were included. Pregnancy was not an exclusion factor.

### Exclusion criteria

Participants undergoing radiotherapy due to head and neck cancer were excluded, as were those with temporomandibular disorders (TMD).

### RPD fabrication

All RPDs were fabricated in the same dental laboratory (Rübeling + Klar Dental-Labor, Berlin, Germany) according to a standardized protocol. The preparation was performed with rotary diamond instruments (Komet Dental, Gebr. Brasseler GmbH & Co. KG, Lemgo, Germany). The controlled circular tooth substance removal was 1.0 to 1.5 mm and the retentive preparation was performed with a preparation angle of approximately 4° to 6° [47]. All tooth impressions were made with polyether material (Impregum, Permadyne, 3 M ESPE, Neuss, Germany). Additionally, all primary crowns were fabricated with a tapered angle of 2° from a cobalt-chromium-molybdenum (Okta-C SAE DENTAL VERTRIEBS GMBH, Bremerhaven, Germany) alloy. Clinically, the internal fit of the primary crowns was checked with light-viscosity silicone (Fit Checker™ Advanced, GC EUROPE N.V., Leuven, Belgium), and the position of the primary crowns on the abutments was transferred into a new master cast using a polyether border molding pick-up impression.

**Fig. 1:**
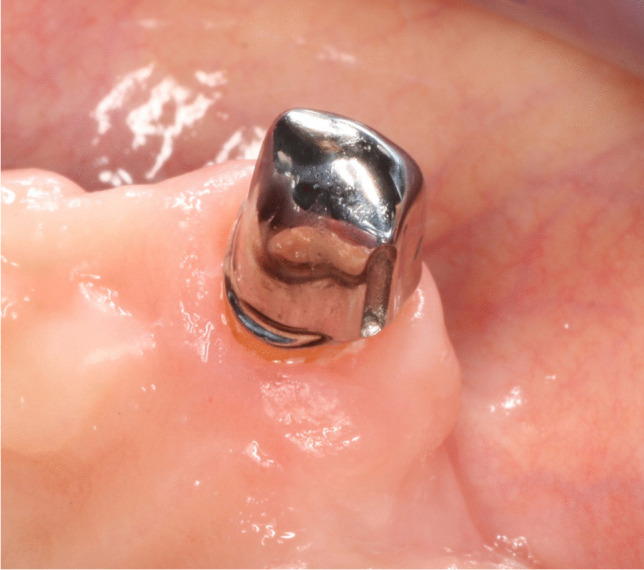
Primary crown with insertion groove (0°) on one approximal surface

**Fig. 2:**
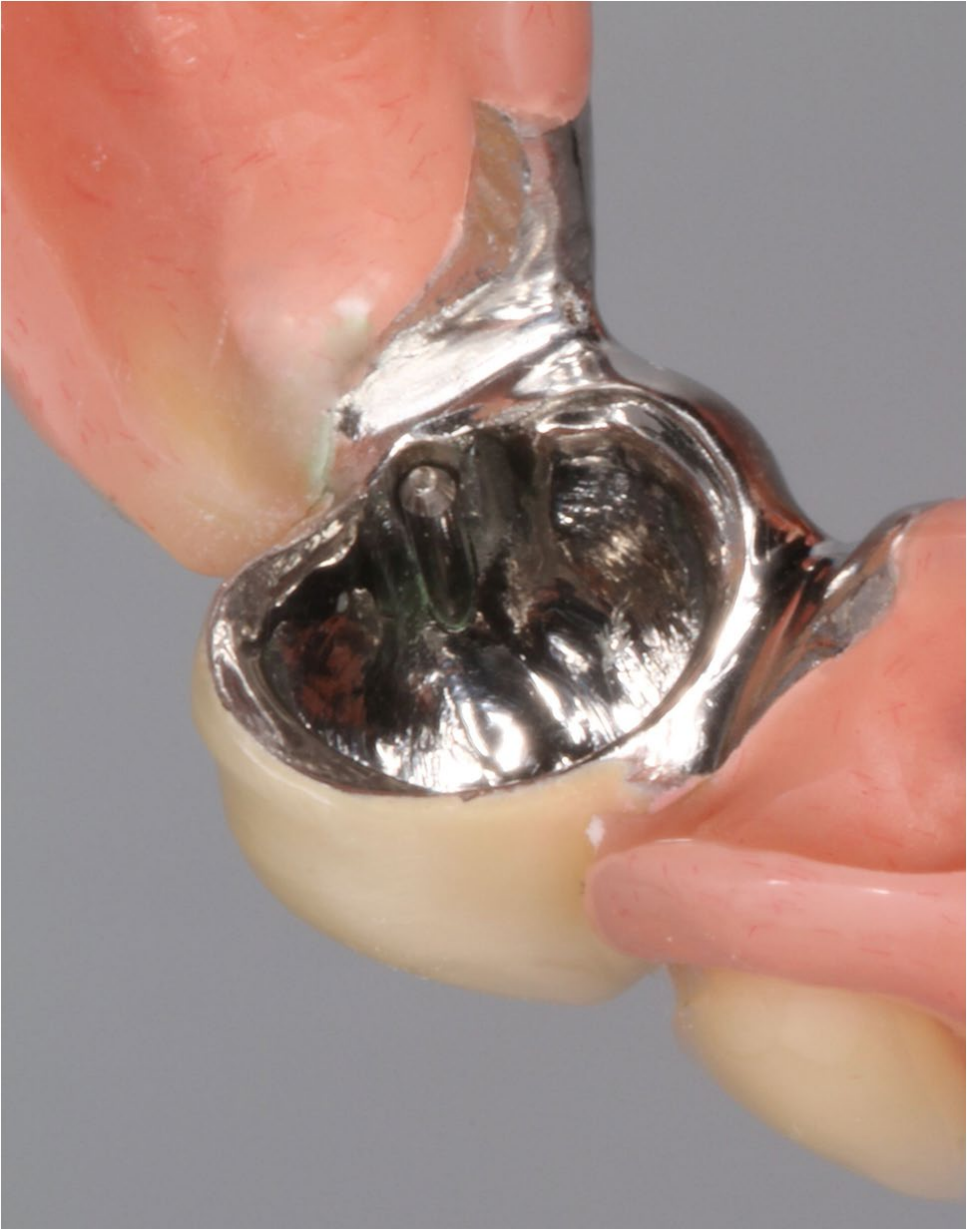
Secondary crown with corresponding friction pins, fixed by laser welding

Subsequently, the manufacture of the denture frameworks and incorporation of the friction pins was performed. A passive fit was achieved by the spark erosion process, in which an insertion groove (0°) was introduced into an approximal surface of the primary crown (Fig. 1). Furthermore, the corresponding friction pin (*Ø* = 0.7–0.9 mm) was fixed in the secondary crown by laser welding (Fig. 2). During the subsequent overall try-in of the dentures, the centric relation, occlusion, framework design, and esthetics were checked.

Special attention was paid to a periodontally hygienic design of the dentures. The definitive placement of all primary crowns was performed with zinc phosphate cement (Hoffmann’s CEMENT normal setting, Hoffmann Dental Manufaktur, Berlin, Germany). All treatment steps were carried out by calibrated practitioners. Finally, all participants were given detailed instructions on the correct handling and care of the dentures after completion.

### Data collection

The retrospective data collection was based on the participant chart and anonymized. The following data were collected: age, sex, date of insertion of the denture, date of the last dental check-up, supplied jaw, denture classification according to Steffel, the position of the abutment teeth according to the FDI (*Fédération Dentaire Internationale)* scheme, the vitality of the abutment teeth, number of double crowns per denture, number of lost retaining elements, number of relinings, number of activations of the friction pins, and date and reason of loss of function of the dentures and the abutment teeth. A determination of the sample size was not performed. All patients fitting the profile (treatment with double crown-retained RPD with friction pins) who received prosthodontic treatment care between 2006 and 2022 were included in the study. Only patients for whom clinical follow-up was available were considered. Accordingly, three additional patients who had received an RPD of interest during the indicated period but never returned for follow-up were not included.

### Follow-up

For participants with this kind of prosthesis, the interval for follow-up was set at 6 months. Further follow-up visits were scheduled according to the individual circumstances of the participants. The 6-month routine follow-up examinations were performed by trained and calibrated practitioners of the Department of Prosthetic Dentistry of the University School of Dental Medicine of the Martin-Luther-University Halle-Wittenberg.

### Statistical analysis

Depending on the number of remaining abutment teeth, participants were subdivided into two groups: (i) more than 3 teeth, non-severely reduced dentition (NSRD), (ii) less than or equal to 3 teeth, severely reduced dentition (SRD). In the SRD group, the distribution of abutment teeth was further subdivided according to Steffel classification [23, 26] for further evaluation: Class A = one remaining tooth with punctual support, Class B = two remaining teeth with linear sagittal support, Class C/D = two remaining teeth with linear transversal/diagonal support, and Class E = three remaining teeth with triangular support.

RPD survival was defined as the time from when the definitive prosthesis was inserted to the day when the functional loss occurred, either due to total loss of abutment teeth or technical deficiencies that could not be corrected to restore the function of the dentures.

Abutment teeth were considered as surviving until the day of loss of function due to extraction (EX) or decapitation (DX).

Cumulative survival at 120 months was calculated using the Kaplan–Meier method. The confidence interval was set at 95% [48].

The influence of variables such as age, sex, jaw, dentition status (SRD vs. NSRD), abutment tooth type, abutment tooth vitality, need for prosthesis relining, primary crown recementation, and reactivation of friction on the long-term survival of both RPDs and abutment teeth was examined over 120 months using the log-rank test and/or Cox regression. Significance was set at *α* = 0.05 for all analyses. A post hoc power analysis for differences in survival between SRDs and NSRDs was performed for both RPDs and abutment teeth. All calculations were performed using the IBM SPSS 28 statistical software (IBM Incorp., Armonk, USA).

## Results

### RPD and abutment teeth characteristics

Of the 520 (100%) abutment teeth, comparable numbers were located in the maxilla (*n* = 262; 50.38%) and mandible (*n* = 258; 49.62%). Canines were the most frequent abutment teeth (*n* = 220; 42.31%), followed by premolars (*n* = 159; 30.58%), incisors (*n* = 79; 15.19%), and molars (*n* = 62; 11.92%). At denture delivery, 61 (11.73%) of these abutment teeth were endodontically treated. The 182 (100%) RPDs were equally distributed in the maxilla (*n* = 92; 50.55%) and the mandible (*n* = 90; 49.45%).

Overall, the SRD group accounted for the largest proportion of abutment teeth (*n* = 314; 60.38%) and dentures (*n* = 144; 79.12%). The majority of SRD abutment teeth (*n* = 178; 34.23%) and dentures (*n* = 82; 45.05%) were distributed among men (Table 1). The mean participant age in the SDR group was 62.7 ± 12.4 years (range, 24.5 to 87.0).

**Table 1:** Removable partial denture (RPD) and abutment teeth characteristics

RPDs		SRD				NSRD	Total
		Class A	Class B	Class C/D	Class E		
Upper jaw	Women	8	13	7	9	10	47
	Men	10	8	9	9	9	45
	Total	18	21	16	18	19	92
Lower jaw	Women	2	8	8	7	7	32
	Men	10	7	10	19	12	58
	Total	12	15	18	26	19	90
Abutment teeth							
Upper jaw	Women	8	30	14	27	53	132
	Men	10	20	18	27	55	130
	Total	18	50	32	54	108	262
	Incisors	0	18	2	10	38	68
	Canines	10	11	21	16	25	83
	Premolars	3	11	4	16	26	60
	Molars	5	8	5	9	19	46
Lower jaw	Women	2	18	16	21	35	92
	Men	10	16	20	57	63	166
	Total	12	34	36	78	98	258
	Incisors	0	1	0	2	8	11
	Canines	11	13	32	46	35	137
	Premolars	0	21	4	26	48	99
	Molars	1	1	0	7	7	16
Endodontic status	Vital	25	76	62	127	169	459
	Endodontically treated	4	6	2	1	12	25
	Endodontically treated + post	1	2	4	4	25	36
	Total	30	84	68	132	206	520

*SRD*, severely reduced dentition; *NSRD*, not severely reduced dentition; *Class A*, one remaining tooth with punctual support; *Class B*, two remaining teeth with linear sagittal support; *Class C/D*, two remaining teeth with linear transversal/diagonal support; *Class E*, three remaining teeth with triangular support

The NSRD group comprised 206 (39.62%) abutment teeth and 38 (20.88%) dentures. In this group, most abutment teeth (*n* = 118; 22.69%) were found in men, and dentures (*n* = 19; 10.44%) were equally distributed between the two sexes. The mean participant age in the NSDR group was 62.1 ± 14.1 years (range, 26.2 to 81.2).

### RPD survival analysis

The cumulative survival rate of all RPDs after 120 months was 65.5% (CI, 53.9 to 77.1).

In the NSRD group, the RPD cumulative survival rate after 120 months was 95.0% (CI, 85.2 to 100). Only one RPD failed in this group within a mean observation time of 61.6 ± 38.4 months (range, 1.4 to 158.8).

For RPDs in the SRD group, the cumulative survival rate was 57.9% (CI, 44.1 to 71.7), which was statistically significantly lower than that in the NRSD group (*p* = 0.004). Within a mean observation period of 67.2 ± 39.7 months (range, 1.4 to 158.8), 29 (15.93%) dentures were extended to complete dentures (CD) and 6 (3.3%) dentures failed due to technical defects or loss (TD). Post-hoc power analysis showed a power of 95.5% for the difference in survival rates between NSRD and SRD.

**Fig. 3:**
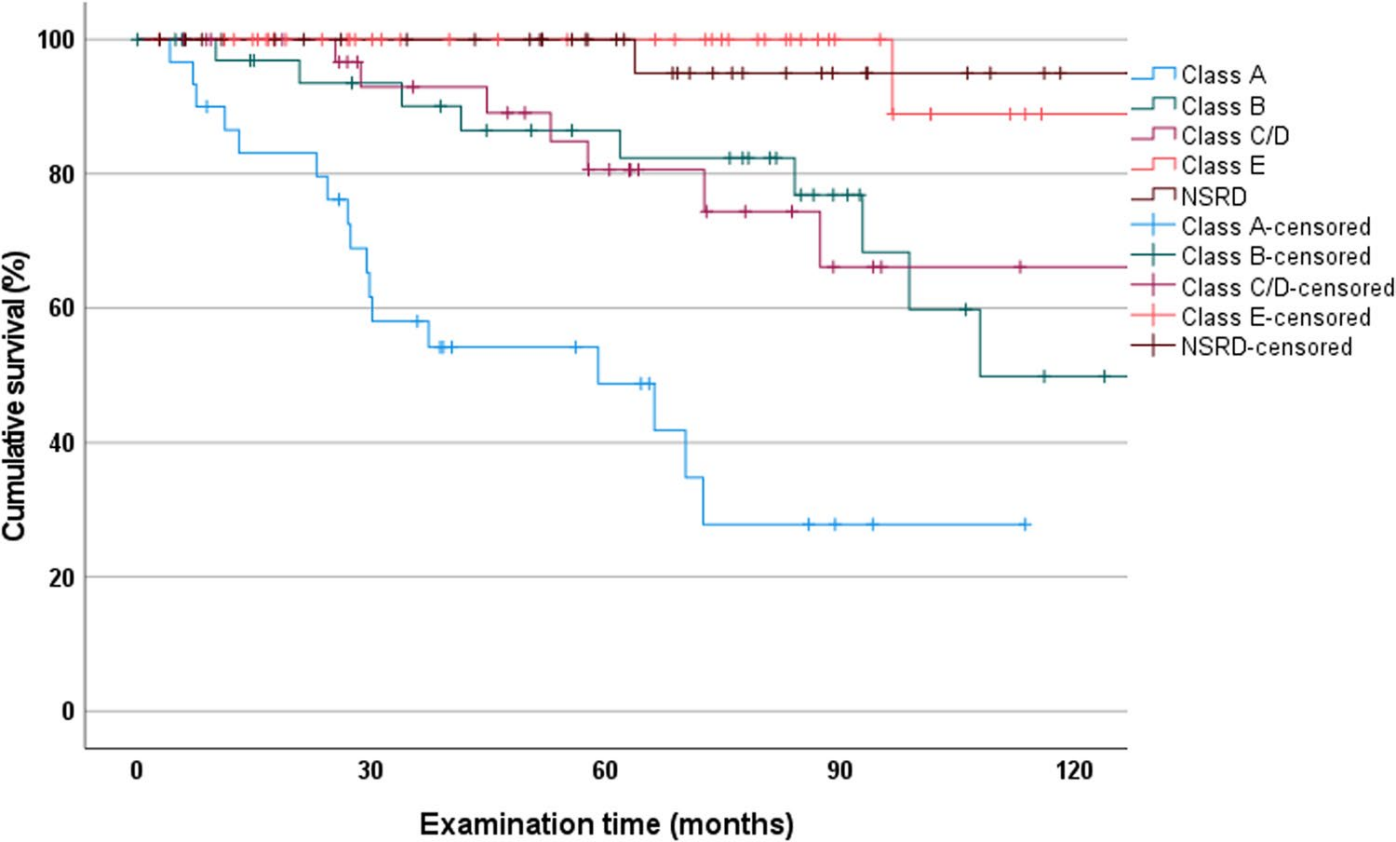
Kaplan-Meier survival curves of RPDs in the NSRD group and Steffel classes. (NSRD = not severely reduced dentition)

Based on Steffel classification, RPDs in the SRD group showed that Class A RPDs failed most frequently (TD, *n* = 2; CD, *n* = 15), followed by those of Class B (TD, *n* = 4; CD, *n* = 5), Class C/D (CD, *n* = 7), and Class E (CD, *n* = 1). Figure 3 shows the survival rates in each subgroup. Class A had a statistically significantly lower survival rate after 114 months than all other classes (27.9%; CI, 6.5 to 49.3; *p* < 0.001). A statistically significant difference in survival between NSRD and class B (49.8%; CI, 22.0 to 77.6; *p* = 0.009) and C/D (66.1%; CI, 43.3 to 88.9; *p* = 0.016) was observed. In contrast, there was no statistically significant difference in survival between NSRD and class E (88.9%; CI, 67.9 to 100; *p* = 0.915).

In the SRD group, participants below and above the median age of 62 years showed an RPD survival rate of 63.3% (CI, 45.9 to 80.7) and 68.7% (CI, 54.5 to 82.9), respectively. However, the difference was not statistically significant (*p* = 0.940). Furthermore, there was no statistically significant difference (*p* = 0.618) between women (68.4%; CI, 51.8 to 85.0%) and men (63.2%; CI, 47.0 to 79.4). Additionally, survival rates between the mandible (65.8%; CI, 46.0 to 85.6) and the maxilla (63.6%; CI, 49.4 to 77.8) were not statistically significant (*p* = 0.151).

Of the initial 182 dentures, 48 (26.08%) were relined within the examination period. Relined dentures had a statistically significantly lower (*p* = 0.052) survival rate of 54.6% (CI, 33.4 to 75.8) than those not relined, which was 70.1% (CI, 56.7 to 83.5).

Within the entire follow-up period, 31 (17.03%) of the 182 RPDs retention was reactivated via the friction pins. However, this did not have a statistically significant effect on the survival of the dentures. Survival rates for reactivated and non-reactivated dentures were 67.8% (CI, 46.8 to 88.8) and 64.1% (CI, 49.9 to 78.3) (*p* = 0.984), respectively.

### Abutment teeth survival analysis

After 120 months, the cumulative survival rate of all abutment teeth was 65.6% (CI, 59.0 to 72.2). A total of 79 (25.2%) teeth were lost in the SRD group and 32 (15.5%) teeth were lost in the NSRD group. The detailed distribution of lost abutment teeth is shown in Table 2.

**Table 2:** Characteristics of abutment teeth failures

Abutment teeth		Intervention	Fracture		Periodontitis		Endodontic problems		Caries		Not specified		Total	
			NSRD	SRD	NSRD	SRD	NSRD	SRD	NSRD	SRD	NSRD	SRD	NSRD	SRD
Maxilla	Incisors	EX	4	6	0	1	1	3	1	0	1	1	7	11
		DX	1	0	0	0	0	0	0	0	0	0	1	0
	Canines	EX	3	13	0	3	1	1	0	2	0	2	4	21
		DX	1	2	0	0	0	0	0	1	0	0	1	3
	Premolars	EX	1	3	2	2	1	1	2	0	0	1	6	7
		DX	0	0	0	0	0	0	0	0	0	0	0	0
	Molars	EX	2	1	1	2	1	0	1	1	0	0	5	4
		DX	0	0	0	0	0	0	0	0	0	0	0	0
Mandible	Incisors	EX	0	0	0	0	0	0	0	0	0	0	0	0
		DX	0	0	0	0	0	0	0	0	0	0	0	0
	Canines	EX	0	9	1	8	0	3	0	1	0	1	1	22
		DX	1	2	0	0	0	0	0	0	0	0	1	2
	Premolars	EX	0	4	0	2	3	1	0	0	0	0	3	7
		DX	2	1	0	0	0	0	0	0	0	0	2	1
	Molars	EX	1	1	0	0	0	0	0	0	0	0	1	1
		DX	0	0	0	0	0	0	0	0	0	0	0	0
Total		EX	11	37	4	18	7	9	4	4	1	5	27	73
		DX	5	5	0	0	0	0	0	1	0	0	5	6

*EX*, extraction; *DX*, decapitation

**Fig. 4:**
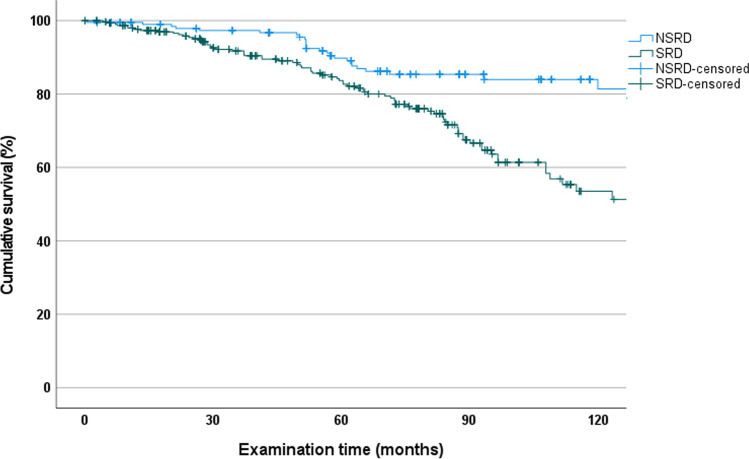
Kaplan–Meier survival curves of abutment teeth in the NSRD and SRD groups. (NSRD = not severely reduced dentition; SRD = severely reduced dentition)

In the NSRD group, the cumulative survival of abutment teeth was significantly higher (*p* < 0.001) at 81.4% (CI, 73.6 to 89.2) than in the SRD group at 53.5% (CI, 43.9 to 63.1) (Fig. 4). Post-hoc power analysis showed a power of 76.1% for NSRD and SRD.

**Fig. 5:**
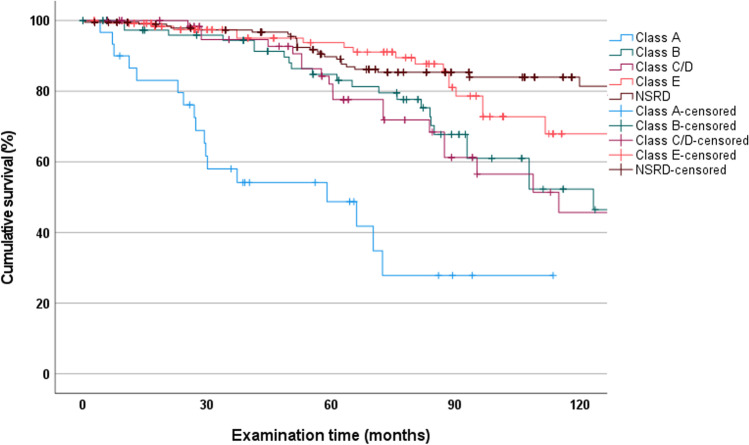
Kaplan–Meier survival curves of abutment teeth in the NSRD group and Steffel classes. (NSRD = not severely reduced dentition)

Subdivided by Steffel classes, abutment-tooth losses were recorded in the SRD group as follows: Class A, *n* = 17 (EX, *n* = 14; DX, *n* = 3); Class B, *n* = 23 (EX, *n* = 22; DX, *n* = 1); Class C/D, *n* = 20 (all EX); Class E, *n* = 19 (EX, *n* = 17; DX, *n* = 2). The effect of abutment tooth distribution on tooth survival was statistically significant (*p* < 0.001) (Fig. 5). In addition, Class E had the highest abutment tooth survival (67.9%; CI, 52.7 to 83.1), followed by Class B (52.3%; CI, 35.1 to 69.5), Class C/D (45.7%; CI, 25.9 to 65.5), and Class A (27.9%; CI, 6.5 to 49.3). A statistically significant difference between classes E and A (*p* < 0.001) and C/D (*p* = 0.023) was observed. In contrast, no statistically significant difference in the survival rates of the abutment teeth was observed between class E and B (*p* = 0.063).

Additionally, the sex of the participants had a statistically significant influence on the survival of the abutment teeth. As a result, abutment teeth in men demonstrated a higher survival rate of 69.2% (CI, 60.6 to 77.8) than in women at 61.1% (CI, 50.3 to 71.9) (*p* = 0.043).

Additionally, in the NSRD group, the survival rate for abutment teeth was statistically significantly higher (*p* = 0.004) for men at 82.5% (CI, 71.5 to 93.5), than for women at 67.8% (CI, 49.0 to 86.6). As opposed to this, the SRD group showed no significant difference in survival rates for abutment teeth in women (59.0%; CI, 47.0 to 71.0) than in men (46.0%; CI, 30.6 to 61.4) (*p* = 0.976).

Based on all abutment teeth, tooth type had no effect on survival (*p* = 0.079). Among the subgroups, tooth type had a statistically significant effect on survival in the SRD group (*p* = 0.016), while no statistically significant influence could be detected in the NSRD group (*p* = 0.473). Additionally, in the SRD group, tooth type-specific survival rates were as follows: molars, 77.7% (CI, 59.1 to 96.3); premolars, 70.1% (CI, 55.1 to 85.1); canines, 41.2% (CI, 25.8 to 56.6); and incisors, 34.6% (CI, 5.6 to 63.6). In the NSRD group, survival rates were as follows: molars, 80.9% (CI, 63.5 to 98.3); premolars, 78.7% (CI, 62.3 to 95.1); canines, 81.4% (CI, 67.2 to 95.6); and incisors, 85.2% (CI, 74 to 96.4).

The survival rate of the abutment teeth differed statistically significantly depending on their location in the maxilla or the mandible (*p* = 0.041). Abutment teeth in the mandible showed a higher survival rate (77.2%; CI, 63.2 to 81.2) than those in the maxilla (61.0%; CI, 52.2 to 69.8). For the subdivision into the NSRD and SRD groups, the values were as follows. Maxillary abutment teeth in the NSRD group showed survival of 76.7% (CI, 65.5 to 87.9) and for mandibular abutment teeth of 89.0% (CI, 81.6 to 96.4) (*p* < 0.001); in the SRD group maxillary abutment teeth showed a survival rate of 48.7% (CI, 36.5 to 60.9) and mandibular abutment teeth of 60.9% (CI, 47.3 to 74.5) (*p* < 0.001).

Relining the dentures had a statistically significant effect on the survival of the abutment teeth. Teeth in dentures with and without relining had survival rates of 44.1% (CI, 28.5 to 59.7) and 73.0% (CI, 66.0 to 80.0) (*p* < 0.001), respectively. In the NSRD group, there were no statistically significant differences in abutment teeth survival rates with (68.1; CI, 42.5 to 93.7) and without relining (83.9.0%; CI, 76.1 to 91.7) (*p* = 0.482). In the SRD group, however, there were statistically significant differences, with abutment teeth of relined RPDs showing 40.4% (CI, 24.6 to 56.2) and those of non-relined RPDs showing 61.0% (CI, 50.1 to 73.3) survival (*p* = 0.012).

The survival rate of abutment teeth of participants below the medium age of 62 years was 69.8% (CI, 60.4 to 79.2), while those above this had 62.4% (CI, 53.4 to 71.4) (*p* = 0.067).

Vital abutment teeth (66.9%; CI, 59.7 to 74.1) showed statistically significantly higher survival rates than endodontically treated teeth (58.2%; CI, 43.6 to 73.4.5) (*p* = 0.001).

In the SRD group, vital abutment teeth had statistically significantly higher survival rates (56.2%; CI, 46.0 to 66.4) than endodontically treated teeth (35.2%; CI, 13.4 to 57.0) (*p* < 0.001).

A similar pattern was found for abutment teeth in the NSRD group: vital, 82.6% (CI, 73.8 to 91.4); endodontically treated, 75.8% (CI, 59.8 to 91.8) (*p* = 0.030).

A total of 33 (6.3%) primary crowns were displaced. All crowns could be successfully recemented. The survival rate of abutment teeth with non-recemented crowns was 63.6% (CI, 63.0 to 76.2) which was significantly higher compared to 11.7% of recemented crowns (CI, 0 to 32.3) (*p* < 0.001).

Within the entire follow-up period, 83 (16.0%) double crown attachments were reactivated via the friction pins. However, this did not have a significant effect on the survival of the abutment teeth. For reactivated abutment teeth, the survival rate was 61.6% (CI, 48.6 to 74.6) and for non-reactivated abutment teeth, 66.8% (CI, 59.0 to 74.6) (*p* = 0.453).

### Multivariate analysis

The estimated hazard ratios and 95% confidence intervals of the multivariate analysis are shown in Table 3. Dentition status and steffel classification had a significant effect on RPD survival. There was a statistically significant effect of age, jaw, endodontic status, dentition status, and steffel classification on the survival of abutment teeth.

**Table 3:** Hazard ratios of the different variables included in multivariate analysis

RPDs				
Variable		Hazard ratio	95%-CI	*p*-value
Age		1.025	0.996 to 1.056	0.089
Sex	Men vs. women	0.661	0.335 to 1.306	0.234
Jaw	Maxilla vs. mandible	0.506	0.250 to 1.024	0.058
Dentition status	NSRD vs. SRD	12.144	1.646 to 89.602	0.014
SRD	Reference NSRD			< 0.001
	Class A	37.460	4.950 to 283.503	< 0.001
	Class B	9.434	1.195 to 74.498	0.033
	Class C/D	8.732	1.074 to 71.004	0.043
	Class E	0.944	0.059 to 15.105	0.968
Abutment teeth				
Variable		Hazard ratio	95%-CI	*p*-value
Age		1.029	1.013 to 1.046	< 0.001
Sex	Men vs. women	1.255	0.853 to 1.845	0.248
Jaw	Maxilla vs. mandible	0.635	0.426 to 0.947	0.026
Endodontic status	Vital vs. endodontically treated	2.321	1.424 to 3.783	< 0.001
Dentition status	NSRD vs. SRD	2.155	1.404 to 3.308	< 0.001
SRD	Reference NSRD			< 0.001
	Class A	8.750	4.782 to 16.010	< 0.001
	Class B	2.032	1.188 to 3.477	0.010
	Class C/D	2.333	1.330 to 4.091	0.003
	Class E	1.070	0.655 to 2.092	0.595
Tooth type	Reference molars			0.085
	Incisors	1.177	0.559 to 2.479	0.667
	Canines	1.610	0.841 to 3.080	0.151
	Premolars	0.912	0.450 to 1.848	0.799

*RPD*, removable partial denture; *SRD*, severely reduced dentition; *NSRD*, not severely reduced dentition; *Class A*, one remaining tooth with punctual support; *Class B*, two remaining teeth with linear sagittal support; *Class C/D*, two remaining teeth with linear transversal/diagonal support; *Class E*, three remaining teeth with triangular support

## Discussion

The null hypothesis was rejected as the 10-year survival rates of both RPDs and abutment teeth in SRDs and NSRDs were different. The variables age, jaw, dentition status (SRD vs. NSRD), abutment tooth type, and abutment tooth vitality showed a significant influence on the survival probability. This study aimed to investigate the long-term survival of DCP-RPDs and abutment teeth after 120 months and to analyze factors that might have an impact on survival rates. To the authors’ knowledge, there is only one other survival study dealing with non-precious metal double crown-retained prostheses with spark-eroded friction pins assessing 60-month survival [23].

Some meta-analyses exist on the survival rates of double crowns [19, 28, 49]. The individual studies vary considerably in terms of follow-up duration, the number of participants, type of double crowns, and cohort, which in turn limits comparability. A study did not name the double crown type that was investigated [50].

Studies that investigate wearing periods of 10 years or more are found less frequently; shorter periods are usually considered [6, 20, 29]. The number of participants in the present study was comparable to other clinical studies investigating other double crown types [24, 51, 52].

In this study, after a follow-up period of 120 months, the survival rate for all RPDs was 65.5%. Several meta-analyses found similar high survival rates for double crown-retained RPDs. However, it should be noted that these meta-analyses included other types of double crowns and, in some cases, shorter study periods [6, 21, 28, 49, 53].

In these studies, higher survival rates may be due to a larger proportion of treatment cases with a more favorable abutment teeth distribution. The proportion of vital and endodontically treated abutment teeth also had a decisive influence on the survival rate of the dentures [54, 55]. Therefore, a lower proportion of endodontically treated abutment teeth could be responsible for better results. However, in some cases, these parameters were not consistently reported in individual studies.

In the present study, as in other studies, it was shown that biological complications were the main reason for the failure of abutment teeth [2, 49, 56]. The most frequent cause was the fracture of an abutment tooth.

Additionally, RPD failure may be defined differently based on different studies. In the present study, both the remodeling of RPDs into complete dentures (*n* = 29) and the damage or loss of the denture (*n* = 6) were classified as a failure. The survival rate of the prostheses would likely have increased if remodeling into total dentures had not been accounted for as prosthesis failure in the present study.

Compared to the 60-month survival analysis, a decrease in survival of 18.8% could be noted [23].

After 120 months, the RPDs in the NSRD group showed statistically significantly better survival than those in the SRD group. This is most likely due to the larger number and more favorable distribution of abutment teeth, resulting in better support of the dentures and more favorable force dissipation. This trend was already evident in the 60-month survival analysis [23], which was later confirmed [19, 20, 28].

The survival rates of Steffel Class A-E dentures were all below those of the NSRD group. Only class E dentures came close to these survival rates. This could be due to a more even and homogeneous distribution of forces in daily use with triangular-supported dentures, which better prevent the overloading of individual teeth.

This supports the finding of other studies that the prognosis of double crown RPDs could be correlated with the number and distribution of abutment teeth [6, 20, 25].

In addition, a Multivariate analysis was performed to weigh the influence of the studied parameters in relation to the survival of DCP-RPDs. Again, in agreement with the results already discussed, it was found that the survival of dentures depends on the distribution of abutment teeth. Steffel class E achieved the best results in the SRD group. Additionally, age, sex, and jaw had no significant effect on denture survival.

After 120 months, the abutment tooth survival rate was 65.6%, which is comparable to other double crowns with additional retention elements (Marburger double crowns) [5, 19, 20, 29, 30, 53]. Compared to the results of the 60-month follow-up, a decrease in the survival rate of 17.8% was observed [23].

In the NSRD group, the survival rate of abutment teeth was statistically significantly higher than in the SRD group. In the literature, similarly increased abutment teeth survival data for different kinds of double crowns were found [6, 29, 56]. However, they did not use the Steffel classification to characterize the SRD group.

In the SRD group, the survival rates of the abutment teeth were lower than those of the NSRD group. As expected, Class A showed the lowest survival rates for abutment teeth; whereas, Class E had the highest. Class B showed better rates than C/D. Comparable survival rates were found in the literature for telescopic crowns [25]. The study design was similar to the study presented here. Its authors suggested that the survival rates of Class A abutment teeth might be due to regular and thorough follow-up. All other Steffel classes (B-E) showed better long-term results, which correlated with the number and distribution of the abutment teeth. However, the increased risks of SRD must be seen in relation to the unfavorable conditions of the patients to be treated and the benefit that they may also have with a shorter clinical application compared to better conditions.

Despite using different types and materials of double crowns, the results of the studies were comparable, suggesting that the types of double crowns had little influence on the survival rates [25].

The type of tooth had no significant influence on the survival rate of all abutment teeth (NRSD and SRD). Other studies have demonstrated this influence [20, 56]. However, when the SRD subgroup was considered in isolation, tooth type showed a significant influence. Here, molars achieved the highest survival rates, followed by premolars, canines, and anterior teeth. This is consistent with results from the previous literature [25].

The higher survival rate of the molars could be due to the fact that they are usually integrated into a denture in combination with other tooth types, which in turn automatically leads to better support of the prosthesis (increasing Steffel class). Canines, on the other hand, are often present as the last abutment teeth when all other teeth are lost. Therefore, they may have to withstand greater leverage of the dentures.

Multivariate analysis was performed to weigh all the parameters studied in terms of their influence on the survival of the abutment teeth. Factors such as age, sex, endodontic status, and teeth distribution also had a significant influence on the survival of abutment teeth. When interpreting the results of the multivariate analyses, it should be noted that the calculated values are associated with wide 95% confidence intervals. This in turn is due to the small number of abutment teeth and DCP-RPDs included in the individual Steffel classes. Overall, this reduces the relevance of the values.

In general, the risk of losing teeth increases when they are non-vital [57]. This was also found in the present study, where vital abutment teeth showed statistically significantly higher survival rates than endodontically treated teeth. In quite a few other studies, it has been found that the survival rate of vital abutment teeth can be up to twice as high compared to non-vital teeth [6, 25, 55, 58]. In contrast, Yoshino et al. found no negative effect of non-vital abutment teeth on survival [20].

However, concerning the mandibular parameter, Szentpétery et al. also found that telescopic crowns in mandibles had a higher survival rate [25]. This is consistent with the results of our study and is ultimately understandable, since the mandible has a more compact structure than the maxilla. Therefore, the last remaining teeth are found far more frequently in the mandible. In our 60-month survival analysis, this parameter was not yet significant. Advanced age is normally associated with an increased risk of tooth loss [59, 60]. In the present study, the age of the participants had a significant effect. Others also found a significant effect of age [20]. On the other hand, some studies could not prove a significant effect [56].

This study had several limitations. First, dental treatment was performed by a variety of dentists with different clinical experiences, but all RPDs were manufactured following the same procedures in a single dental laboratory. Second, the post-treatment and fitting procedures were performed by different dentists. Nonetheless, all of them adhere to standards agreed to by the department where the study was conducted, which may very well reflect clinical practice reality.

Third, despite the 6-monthly check-ups being standard, patients deviated from this due to non-compliance. This can be quantified as about 35% of the patients considered. Wöstmann et al. found that a regular recall system increased the survival rate of abutment teeth [6]. Thus, if regular check-ups and follow-up treatments had been performed, the survival rate of abutment teeth might have improved.

Fourth, since the data were analyzed retrospectively based on patient records, only documented events could be included in our analysis. Finally, the restoration of the opposing jaw, unless it was also a DCP-denture, was not considered in this study. Different masticatory forces can be generated with different dentures. The restoration in the opposing jaw can also change several times during such a long follow-up period. This could have been a confounding factor that was not considered in our analysis.

Fifth, the periodontal condition of the abutment teeth was not specifically considered, although. only those teeth that were judged to be periodontally healthy were used as abutment teeth. Periodontal therapy was performed during the follow-up period if considered to be necessary.

Sixth, the distribution of abutment teeth and prostheses (SRD and NSRD) was not similar in both groups. This could have led to a bias of the results. Ideally, both study groups would be of equal size. However, this is not possible to define in advance in the context of a clinical retrospective follow-up. Overall, the sample size in both groups appears to be sufficient compared to other studies [24, 51, 52].

If abutment teeth are lost, DCP-RPDs can be easily extended to full dentures. Overall, the DCP-RPDs can be equated with other double crown systems. However, the possibility of simply activating the dentures by the friction pin can be easily performed chair side without spare parts. This is a great advantage compared to any other double crown system.

## Conclusion

After 120 months, DCP-RPDs showed acceptable survival rates in both the SRD and NSRD groups. The number and distribution of abutment teeth per RPD seemed to be the main determinant of long-term success. Thus, the NSRD group showed the lowest overall failures. In the SRD group, Steffel class E showed the lowest failures and statistically no difference to the NSRD group. Steffel class A with only one abutment tooth showed the worst survival rates. This class could therefore be considered as an orderly transition towards full dentures. Therefore, prosthetic planning should also include sufficiently endodontically treated teeth for abutment augmentation; these could optimize the topographical abutment distribution and therefore contribute to a better survival of the RPD.
